# Madison County Medical Association

**Published:** 1903-05

**Authors:** A. H. Speer

**Affiliations:** Madisonville, Texas


### Madison County Medical Association.

MADISONVILLE, TEXAS, April 26, 1903.

*Texas Medical Journal, Austin, Texas.*

GENTLEMEN: The Madison County Medical Association met in regular session Tuesday, April 14, 1903, and the following officers were elected for the ensuing year: Dr. J. D. Jordan, President; Dr. B. F. Gibson, Vice-President; Dr. A. H. Speer, Secretary; Dr. I. Rasco, Treasurer. Dr. Gibson was elected delegate to the State Convention at San Antonio.

Respectfully,

A. H. SPEER.

